# Avian Liver: The Forgotten Organ

**DOI:** 10.3390/ani9020063

**Published:** 2019-02-15

**Authors:** Faegheh Zaefarian, Mohammad Reza Abdollahi, Aaron Cowieson, Velmurugu Ravindran

**Affiliations:** 1 Monogastric Research Centre, School of Agriculture and Environment, Massey University, Private Bag 11 222, Palmerston North 4442, New Zealand; M.Abdollahi@massey.ac.nz (M.R.A.); V.Ravindran@massey.ac.nz (V.R.); 2 DSM Nutritional Products, Wurmisweg 576, CH-4303 Kaiseraugst, Switzerland; aaron.cowieson@dsm.com

**Keywords:** liver functions, liver metabolism, feed restriction, anti-nutritional factors, feed processing techniques, poultry

## Abstract

**Simple Summary:**

The liver is a multi-purpose organ, with involvement in bile secretion, and lipid, carbohydrate and protein metabolism, as well as a number of other metabolic functions. This organ can adapt easily to changes in feed and the environment. Being at the centre of a number of digestive, metabolic and productive activities, it is essential to have a better understanding of this organ and the factors affecting liver functionality.

**Abstract:**

Despite having huge responsibilities in avian species, published reports on the influence of dietary factors and other possible constraints on the size, development and function of liver are limited. Consideration of the factors that could influence and alter liver function is therefore of critical relevance. In the current review, aspects of liver structure and function, and the influence of feed restriction, anti-nutritional factors, structural components and feed additives on liver are discussed. Effects of feed technology techniques such as thermal treatment and pelleting, feed particle size and whole grain feeding on the liver are also reviewed. A discussion of lipogenesis and lipid storage in poultry is presented to provide a better understanding and to differentiate the normal pathways of lipid metabolism from abnormal (i.e., disordered) pathways. The liver is the main site of fat synthesis in poultry, but under certain conditions, excessive fat can accumulate in the liver and cause problems. Factors contributing to the fatty liver syndrome are also examined.

## 1. Introduction

Similar to mammals, the liver in birds is involved in an array of metabolic and homeostatic functions and considered as a biochemical factory responsible for most of the synthesis, metabolism, excretion, and detoxification processes. It plays an important role in digestion and metabolism, regulating the production, storage, and release of lipids, carbohydrates and proteins [1]. The liver produces a variety of proteins, including blood proteins, enzymes, hormones, clotting and immune factors. It functions as both an endocrine and exocrine gland [1]. In order to maintain a healthy bird, this organ should be kept in an excellent condition. Better understanding of the metabolic functions and the factors that can cause disruptions in the liver is important for the production of heathy birds.

It is the intention of this paper to review the available literature on the constituents, structure and metabolic functions of the liver and to provide an overview of dietary factors, which can influence the liver function in poultry. The focus will be on the influence of (i) feed restriction, (ii) anti-nutritional factors, (iii) structural components (iv) feed additives, and (v) feed processing techniques. Moreover, metabolic disorders related to the liver dysfunction will also be considered. 

## 2. Structure and Functional Units in Avian Species

The liver is an accessory organ of the digestive system and the largest gland of the body. The liver, located in the anterior end of the body cavity, has its conformation modified to fit the contour of the internal surfaces of the body wall, as well as the adjacent and enclosed structures, such as the heart and pericardial cavity, the underlying gizzard, spleen, gall bladder, the loop of the intestine and the lungs [2]. It is divided into two lobes, namely right and left, which are joined cranially at the midline. The right lobe is larger and separated from the left lobe by a deep fissure. In the domestic fowl and turkey, the left lobe is subdivided into the dorsal and ventral parts [1,2]. Visceral peritoneum covers the liver and closely adheres to its surface. There are several attachments and ligaments, such as triangular ligaments and lesser omentum holding this organ in place. Lobules, the functional unit of liver, are hexagonal in shape. Each lobe of the liver has approximately 100,000 lobules. Lobules are separated from each other by interlobular septum. The liver lobule is formed by parenchymal cells (hepatocytes) and non-parenchymal cells. Hepatocytes occupy almost 80% of the total liver volume and perform numerous liver functions. Non-parenchymal liver cells are localised in the sinusoidal wall. Two types of the cells compose the sinusoids, namely endothelia cells that coat the sinusoids and macrophages. The latter are referred to as Kupffer and are star-shaped and confined to the liver. Kupffer cells phagocytise pathogens, cell debris and, damaged red and white blood cells [3]. Sinusoids allow large plasma proteins from the bloodstream into the spaces surrounding the hepatocytes. 

**Figure 1:**
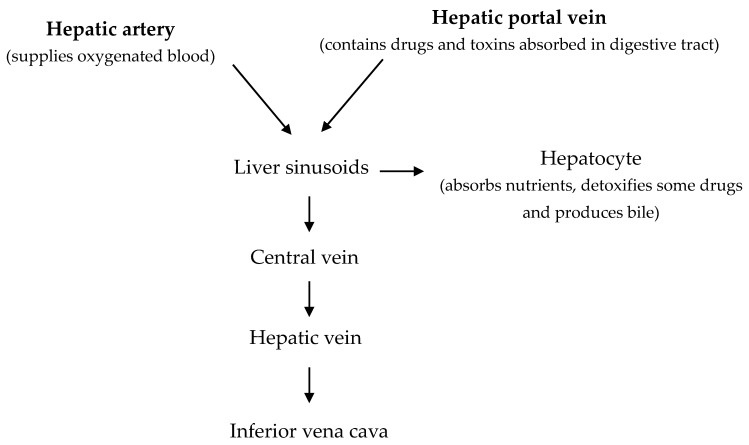
Blood flow through the liver lobule (Adapted from Akers and Denbow [5]).

**Figure 2:**
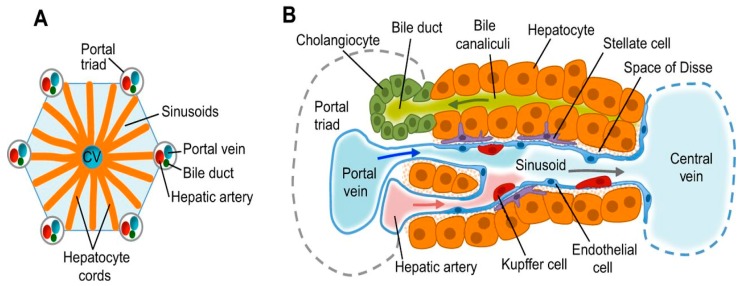
Liver structure and cell types (**A**) Functional unit of the liver lobules; Each lobule is composed of a central vein (CV), the portal triad consists of a portal vein, biliary duct and hepatic artery. Hepatocyte cords are separated by sinusoids that carry blood from the portal triads to the central vein. (**B**) Hepatocytes secrete bile salts into the bile canaliculi that lead to the bile duct. Stellate cells are in the space of Disse between the hepatocyte cords and sinusoids. Kupffer cells, which are the specialised macrophages of the liver, are also located in sinusoids. Epithelial cells lining the bile ducts are called Cholangiocytes (Adapted from Gordillo et al. [6]).

The hepatocyte is a complex cell with a large nucleus and many mitochondria which contain granules. Lysosomes, rough and smooth endoplasmic reticulum, Golgi apparatus, and other organelles are also found in the hepatocyte [4]. Hepatocytes are arranged in plates that radiate longitudinally outward from central vein. At each corner of the hexagonal lobule, there is a portal triad consisting of a branch of the hepatic artery, a branch of portal vein and the bile duct. The liver receives blood from the intestine and the general circulation [5]. The liver receives oxygenated blood from the hepatic artery and deoxygenated blood from the hepatic portal vein, which contains nutrients, drugs and toxins from the digestive tract. Branches from both arteries enter the liver sinusoids where the hepatocytes remove some nutrients and toxins. As the blood passes through sinusoids, metabolites from hepatocytes are secreted into the blood. This blood then passes to the central vein and then into the hepatic vein (Figure 1 and Figure 2).

## 3. Composition and Size

The avian liver has much less connective tissue than the mammalian liver and it lacks a true lobular structure [4]. In avian species, the liver is larger than in mammals when compared to body size. The size and colour of the liver depends on age and body weight [1,7]. In 21-day old broiler chickens fed maize-soy diets, the liver comprises approximately 27 g/kg body weight (BW) and protein content of the liver was 690 g/kg liver mass. There were no differences in the contents of protein, fat and ash between soybean oil- and tallow-supplemented diets (Table 1). 

Bowes and Julian [8] compared the liver weights of broilers at 9 and 42 days of age and observed that over this period the relative weight of liver decreased by 35.3 %, from 37.7 to 24.4 g/kg BW, respectively. Nir et al. [9] found an increase in the relative liver weight from 25 g/kg BW at hatch to 46 and 48 g/kg BW on day 8 in egg-type and meat-type chickens, respectively. The relative weights declined thereafter, but the reductions were greater in egg-type than in meat-type chicks. By day 14, the liver weight of meat-type chicks exceeded that of the egg-type chicks. Ravindran et al. [10] reported that the relative weights of the liver in birds fed wheat-based diets increased to day 14 and decreased thereafter. In their study, the relative weights were 35, 38, 43, 33, 30 and 26 g/kg BW at 1, 7, 14, 21, 28 and 35 days post-hatch, respectively. The heavier relative liver size in early age presumably enable birds to metabolised nutrient more efficiently, due to the lower feed intake and endogenous enzyme secretions.

## 4. Liver Functions

The liver is a vital organ that is involved in a wide range of functions including the metabolism of fat, carbohydrate, protein, vitamins and minerals, removal of waste products and detoxification. The liver is the main storage site of fat-soluble vitamins (A, D, K and E) as well as vitamin B12, glycogen, some minerals (Fe and Cu) and is also involved in the activation of vitamin D. The liver is the main site of phagocytosis by Kupffer cells, which destroy aged blood cells and pathogens that may enter via the hepatic portal blood [5].

### 4.1. Fat Metabolism

The liver plays the main role in lipogenesis, providing lipids destined to be used by all tissues and the liver itself [11]. In contrast to mammals, the synthesis of fat in birds is greater in the hepatic tissue and very limited in the adipose tissue [11]. In laying hens, the liver plays a major role in the synthesis and metabolism of fat. Fats that are metabolised in the liver are derived from three main sources: dietary fat, depot fat and fat from de novo fatty acid synthesis (from feed carbohydrates). Similar to mammals, the digestion and absorption of dietary fats in avian species occur in the small intestine [12]. However, due to a poorly developed intestinal lymphatic system in birds, dietary fatty acids are drained directly into the portal blood system (instead of the lymphatic system) as very low-density lipoproteins (VLDL) which are termed portomicrons [13]. Portomicrons are chylomicrons and contain 90% triglycerides which combined with free and esterified cholesterol, lipoprotein and phospholipids [11,14]. From the portal blood system, most of the portomicrons pass through the liver before they reach the rest of the circulation. This unique feature predisposes the birds to fat accumulation in the liver [15]. Liver hepatocytes can store triglycerides from portomicrons as well as metabolise fatty acids to ATP, synthesise lipoproteins and phospholipids or store the released energy in tissues as fat deposits [16]. The transport of triglycerides from the liver into adipose tissues or the oviduct is facilitated by different classes of lipoproteins.

#### 4.1.1. Gall Bladder, Bile Formation and Secretion

The gall bladder, which stores the bile, is a thin-walled muscular green sac found on the ventral surface of liver. The liver is the source of the bile which plays an important role in the emulsification of dietary fats, an essential step in fat digestion and absorption. Bile is produced in the hepatocytes of the liver and secreted into the bile canaliculi. Canaliculi are the intracellular canals between the hepatocytes and transport bile for storage in the gall bladder [17]. In birds, in contrast to mammals, two bile ducts enter the duodenum just below the gizzard exit. The right and left hepatic ducts combine to form a common hepato-enteric duct, which then goes to the duodenum. However, a hepato-cystic duct branches from the right hepatic duct and connects to the gall bladder which, in turn, is drained by the cystico-enteric duct into the duodenum [18]. The bile ducts generally drain into the duodenum at a site very near the pancreatic ducts and occur on the ascending loop of the duodenum. In some avian species (e.g., ostrich and pigeons), the ducts empty into the descending loop of the duodenum [18]. Bile is a major source of fat secretion into the duodenum and account for the observed negative fat digestibility in this segment [12,19,20,21]. Moreover, digesta and bile are shuttled between the gastric region and duodenum via anti-peristaltic refluxes to enhance the enzymatic and mechanical action of digestion. This action may also increase the net concentration of fat in the duodenum [12].

Relatively little is known about biliary secretion in birds due to the complex anatomy in which bile enters the intestine via both hepato-enteric and the cystico-enteric ducts. Bile flow is stimulated by feed [22], presence of bile salts in the blood [23] and cholecystokinin (CCK) secretion [24]. In ruminants, pigs and poultry, there is relatively continuous secretion of bile into the intestine. The sphincter of hepato-pancreatic ampulla (sphincter of oddi) in these species is less well defined. In dogs and cats, a continuous secretion into intestine is unnecessary because these species eat only once or twice a day [25]. Parasympathetic signals traveling along the vagus nerve can stimulate bile production by the liver. Neural and hormonal stimuli can also stimulate bile secretion. The sphincter of oddi controls the entry of bile into the duodenum. The presence of fatty acids, particularly medium and long chain fatty acids in the chyme stimulates duodenal entero-endocrine cells to secrete CCK and cause the contraction of smooth muscle of the gall bladder and relax the sphincter of oddi. Bile is then squeezed into the cystic duct and through the common bile duct [5]. The biliary secretion rate has been reported to be 24.2 uL/min in fasted birds [26].

#### 4.1.2. Bile Composition and Functions 

Bile consists of water, electrolytes, bile acids, bile salts, and neutral fats such as cholesterol, glycerides, phospholipids (e.g., lecithin), bile pigments and some proteins [27,28]. Bile is the main source of endogenous fat and fatty acids and plays an important role as an emulsifier in the digestion and absorption of fat by reducing the tension at the oil-water interface [28]. Bile is slightly acidic in chickens (5.9), ducks (6.1) and turkeys (6.0) [1,5]. Bile activates pancreatic lipase as well as prevents the denaturation of this enzyme when it leaves the surface of emulsified fat droplets [29]. Electrolytes and water in the bile are reabsorbed by the gall bladder epithelium and there is some passive absorption of fat-soluble compounds such as cholesterol. Bile salts become concentrated 10-20-fold in the gall bladder [25]. Cholesterol, the precursor of bile, is first hydrolysed with 7-α-hydroxylase to form cholic and chenodeoxycholic acids to form taurocholic and glycocholic acids, which make them water soluble at the pH of bile. Bile acids are then conjugated with taurine or glycine and secreted as bile salts. During fat digestion, bile salts along with free fatty acids and lecithin combine to form micelles within the intestinal lumen. The polar ends of molecules are arranged outside of the micelle and the non-polar ends face the inside of the micelle where fat-soluble vitamins are found [28].

Bile acids which are synthesised from cholesterol are termed primary bile acids. The conjugation lowers the isoelectric point and forms charged complexes in the bile and in the intestinal lumen, where the pH is about 6.0. This ensures that the bile salts are not passively absorbed in the upper intestine but are available throughout the small intestine for fat digestion and absorption [25]. The bile salts, glycocholate and taurocholate, are readily absorbed throughout the small intestine, with the rate of absorption being higher towards the distal end [30]. This presumably aids the recycling of bile salts to the liver for reuse [24]. Hurwitz et al. [19] estimated that 90% of bile salts are reabsorbed in the jejunum and ileum. Bile salts escaping intestinal absorption enter the hindgut, where they are deconjugated and dehydroxylated by bacteria. The resulting products are known as secondary bile salts and may cause colonic epithelial damage and diarrhoea [25]. 

Endogenous secretion rates of the bile pigments, biliverdin and bilirubin, in chickens have been reported to be 14.7 and 0.9 µg/kg/min, respectively [31]. Excretion rates for total endogenous bile pigments were found to be greater in chickens than in non-avian species. Biliverdin concentrations are higher than those of bilirubin because the chicken has very low concentrations of glucuronyl transferase and biliverdin reductase [24]. The green colour observed in avian excreta is possibly because of biliverdin and the brown colour is likely due to bacterial reduction of biliverdin to bilirubin and subsequent dehydrogenation [32].

In germ-free chickens, only cholic, allocholic, and chenodeoxycholic acids are observed [24]. In conventionally raised chickens, however, other bile acids originating from the intestinal microbiome are also detected. Elkin et al. [33] found that chenodeoxycholyltaurine and cholyltaurine are the predominant bile acids in chickens and turkeys, whereas chenodeoxycholyltaurine and phocaecholyltaurine predominate in ducks. Yeh and Hwang [34] reported that bile from ducks contained a high amount of taurochenodeoxycholic acid, followed by cholic, ursodeoxycholic, chenodeoxycholic, lithochilic, deoxycholic and taurocholic acids. In chickens, a high concentration of glycolithocholic acid was observed, followed by taurocholic, lithocholic, taurolithocholic, chenodeoxycholic and cholic acids (Table 2). Among wild birds, chenodeoxycholic acid was the primary bile acid, with cholic and allocholic acids dominating in carnivorous species [24]. 

Secretion of bile is thought to be limited in young birds, especially during the first week of life, resulting in reduced fat digestion and absorption [28]. In addition, young birds are unable to recycle bile salts efficiently as older birds and this decreases the pool of bile salts, which may also contribute to the poor digestion of fat [35].

#### 4.1.3. Lipogenesis and Lipoprotein Formation

Because poultry feed formulations contain low concentrations of fat (usually less than 50 g/kg), the liver plays an important role in lipogenesis and the conversion of glucose to triglycerides, which can be used by all tissues, including the liver itself [11]. Klasing [36] stated that avian hepatic cells can synthesise saturated fatty acids from non-lipid substrates (i.e., de novo synthesis) and oxidise them to mono- and di-unsaturated fatty acids. Lipogenesis occurs via a series of linked-reactions including glycolysis, citric acid cycle and fatty acid synthesis [37]. Hermier [11] found that lipogenesis in the liver of chickens is high and particularly active in laying hens producing eggs, because of high oestrogen secretion. De novo hepatic fatty acid synthesis depends on the availability of dietary carbohydrate to provide acetyl coenzyme A [11]. 

While the main products of de novo hepatic lipogenesis are triglycerides, the liver is also the major site of phospholipid and cholesterol synthesis. These lipids, along with proteins, are the components of lipoproteins. In birds, apart from portomicrons, which transport lipids from the gastrointestinal tract (GIT) to the liver via the portal circulation, very lowdensity lipoprotein (VLDL), intermediate density lipoproteins (IDLs), low-density lipoprotein (LDL), and high density lipoproteins (HDL) are the four classes of lipoprotein particles that are synthesised and secreted by the liver [38,39]. The classification of lipoproteins is based on density, which varies depending on the proportion of lipids and proteins that they contain [39]. As mentioned above, VLDL are synthesised in the liver and released into the bloodstream, transporting lipids to other tissues. IDL are the residues of VLDL following their metabolism by other tissues, while LDL originate from the metabolism of VLDL and IDL after lipolysis by both lipoprotein lipase and hepatic lipase. HDL, which begin as disc-shaped particles, originate primarily in the liver and presumably also in the intestine. Another type of lipoprotein is vitellogenin, which is synthesised in the liver of laying birds, under the influence of oestrogen during the production phase and exported directly to the ovaries, where it participates in the formation of the egg yolk [39]. Speake et al. [40] reported that approximately 24% dry mass of egg yolk comes from vitellogenin lipoproteins. The VLDL is the major lipoprotein responsible for the transport of lipids from the liver to the oocyte and accounts for 60% of the dry yolk mass. Griffin and Hermier [41] stated that VLDL concentrations rise at the onset of lay, while HDL concentrations are approximately halved [41]. The specific proteins of the lipoprotein are also synthesised in the liver [42]. Triglycerides are preferentially associated with apolipoprotein B into VLDL particles, whereas most of the phospholipids and cholesterol are associated with apolipoprotein A-1 in HDL. During egg production, the synthesis of yolk lipoproteins by the liver is faster than their mobilisation from the hepatocytes, which may increase the liver size and lipid content. Additionally, the rate of the VLDL absorption by ovarian follicles is not as rapid as hepatic release, resulting in 2–10-fold increase in circulating triglycerides [36].

Literature regarding the regulation of lipoprotein synthesis and secretion, and its hormonal regulation in the liver of birds is scant. However, Tarlow et al. [43] reported that insulin increased de novo lipogenesis and VLDL synthesis, whereas thyroxine and glucagon have the opposite effects. 

### 4.2. Carbohydrate Metabolism

In the developing embryo, liver carbohydrates become available by the action of glycogenic or gluconeogenic enzymes [24]. The concentration of glycogen in the liver (mostly) and muscle (to a lesser extent) fluctuates throughout embryonic life. A day after hatching, both the heart and liver glycogen concentrations decrease dramatically to 40 and 16% of pre-hatching values, respectively [44]. Liver glycogen concentrations remain relatively low for several weeks post-hatch and increase to adult levels by 4 months. The plasma glucose concentration in chickens approaches 150–160 mg/dL at hatching and gradually increases in 4–6 weeks to 190–220 mg/dL [24]. Goodridge [45] reported that the activity of Krebs cycle increases throughout the incubation period, especially in the liver, and continues to rise during embryogenesis and early post-hatch growth. 

In general, the liver, with the help of the pancreas, maintains a constant concentration of blood glucose. When blood glucose concentrations are high, the liver converts glucose to glycogen (glycogenesis) and triglycerides so that the energy can be stored until needed. When blood glucose concentrations drop, the liver can break down glycogen to glucose (glycogenolysis) and releases the glucose into the bloodstream. In response to immediate glucose demand, the liver can convert certain amino acids and fats, as well as lactic acid to glucose [5].

### 4.3. Protein Metabolism

The liver is also involved in protein metabolism. Protein synthesis in the liver represents 11% of all protein synthesis in the bird [1]. Dietary proteins are hydrolysed in the intestine to small peptides and free amino acids by the action of proteases and peptidases. These amino acids are absorbed by the enterocytes and passed into the portal vein. They then enter the liver and are transported via systemic circulation to other tissues and organs. If the amino acids are in excess and not utilised for the synthesis of tissue proteins or hormones/enzymes, they will be catabolised by the liver. Hepatocytes remove the amino group (deamination) of amino acids and form ammonia and keto-acids. Ammonia is toxic to birds. The enzymes of the uric acid cycle are found both in the liver and kidney. The predominant organ responsible for uric acid formation is the liver. The kidney synthesises only about 17% of the uric acid found in the urine of birds [46]. The released ammonia is transformed into the uric acid in the liver and excreted through the kidneys. The keto-acids, on the other hand, can be used for ATP synthesis. Hepatocytes also synthesise carbohydrates and fat from certain amino acids, and can synthesise various plasma proteins such as albumin, prothrombin, fibrinogen, and alpha and beta globulin [5]. Blood clotting proteins and proteins involved in immunity (acute-phase proteins and globulins) are also synthesised in liver hepatocytes. 

### 4.4. Vitamin and Mineral Metabolism

The liver is involved in the storage of fat-soluble vitamins A, D, E and K. Vitamin A is stored in the liver and released when required. Vitamin K is utilised in the liver for the formation of prothrombin, an anticoagulant factor needed for blood clotting. Some members of the vitamin B group, especially B1, B2 and niacin are metabolised in the liver, where they may also be stored. Certain minerals (Fe and Cu) are also stored in the liver [5].

The formation of red blood cells is termed as erythropoiesis. Although normal erythropoiesis takes place in the bone marrow, ectopic erythropoiesis can occasionally occur in the spleen and liver [47]. The lifespan of erythrocytes in chickens is 20–30 days; after which, erythrocytes are destroyed in the liver and, the minerals (Fe, Cu and Co) are released and stored in liver for the use by the body tissues. The liver along with the skin and kidneys synthesise 1,25 dihydroxycholecalciferol [5] from vitamin D3. To be used by the body, vitamin D3 must be metabolised following ingestion into 25-hydroxycholecalciferol (25(OH)D3) in the liver and subsequently into its active metabolite 1,25-dihydroxycholecalciferol (1,25(OH)2D3) in the kidneys [48].

### 4.5. Removal of Waste Products and Detoxification

The liver is the major detoxification organ in the body. Toxic substances from the feed, as well as the toxins produced in the body, are detoxified by the liver. The potential toxins include a diverse group of fat-soluble substances—metabolic end products (e.g., ammonia, products of blood cell destruction, bile pigments), contaminants (e.g., pesticides, carcinogens), anti-nutrients (e.g., hydrocyanic acid), chemicals (e.g., heavy metals), additives (e.g., antibiotics) and drugs (e.g., various medications). Depending on their concentration, these can cause varying degree of damage to the health of the bird. During the process of detoxification, through oxidation, reduction, hydrolysis and conjugation, the liver converts these toxins to more polar and water-soluble waste products, which are then eliminated via kidneys and gall bladder [5]. Importantly, the phagocytic action of Kupffer cells is a principal mechanism by which the microorganisms entering the blood are destroyed [1,5].

## 5. Influence of Fat Sources on Liver Metabolism

In poultry, the capacity for lipogenesis is 20-times greater in the liver compared to adipose tissues. As mentioned earlier, the liver plays a dominant role in the deposition and oxidation of fatty acids. Dietary fatty acids in poultry diets are provided by animal or vegetable oils. Hepatic cells in birds are able to synthesise saturated fatty acids from non-lipid substrates by de novo synthesis and oxidise them to mono- and di-unsaturated fatty acids [36]. Birds, however, are not able to synthesise linoleic and linolenic acids, making them dietary essentials. Both dietary and endogenously produced fatty acids are metabolised within chicken hepatocytes from arachidonic acid, which is further de-saturated by hepatocytes to produce prostaglandins and eicosapentaenoic acid, respectively [36]. 

A large body of research has evaluated supplementation of different n-3 fatty acid sources from marine products as well as plant and seed oils rich in α-linolenic acid in poultry diets. Fish meal and marine oils are well known sources of long chain n-3 poly unsaturated fatty acids (PUFA) and can be used to increase n-3 PUFA content in the muscle. Inclusion of fish oil in broiler and laying hen diets has been reported to reduce the concentrations of liver lipids and yolk triacylglycerol and increase the lipid clearance from the liver compared to plant-derived n-3 oils [49,50,51]. 

Dietary fatty acids interact with the liver and modulate the action of enzymes associated with hepatic metabolic pathways [52]. Senkoylu and Dale [53] found that the addition of high-oil-sunflower meal to broiler starter diets depressed weight gain, feed intake and liver lipids, but not the liver weight. It was suggested that the lower liver lipid associated with feeding high-oil-sunflower meal may be due to the inhibition of lipid synthesis because of the high fibre content of the diet. Smink et al. [54] fed birds diets containing sunflower oil, a mixture of hydrogenated sunflower oil and sunflower oil (50:50), palm oil and randomised palm oil. Absolute liver weight was not affected by the fat type. However, liver fat mass tended to be lower in birds fed sunflower oil compared to other groups. Liver monounsaturated fatty acid content in birds fed sunflower oil was lowest, whereas liver PUFA was not affected by dietary treatments. These results are in agreement with those of Pinchasov and Nir [55] who reported a reduced liver fat content at high inclusion level of PUFA in broiler diets. Smink et al. [54] reported a negative correlation (R = 0.5) between PUFA concentration in the liver and enzyme activities of both acetyl-coenzyme A carboxylase and fatty acid synthase. 

## 6. Influence of Feed Restriction 

Visceral organs constitute a high proportion of the total energy requirement of an animal, and the expenditure of energy in these organs is positively correlated with their mass [56]. Increased liver size (and hence its metabolic activities) due to particular dietary treatments indicate increased utilisation of dietary energy for maintenance at the expense of growth. Pearson et al. [57] suggested that the increased activity of hepatocyte enzymes results in hepatic hyperplasia and hypertrophy, leading to increased liver size. Stangassinger and Giesecke [58] suggested that the increased liver weight was due to cell proliferation as well as to enlargement. It has been reported that early dietary access of newly hatched chicks not only boosts early feed intake and growth, but also increases the relative growth of internal organs such as the small intestine, liver and pancreas [59]. Canas et al. [60] noted that the weights of liver, heart and intestine of rats were functions of feed intake and that the relative weight of these organs were greater in lactating than in non-lactating rats. They further suggested that 24% of the increase in maintenance energy expenditure during lactating animals could be explained on the basis of observed changes in relative weights of these organs. On other hand, Denbow [1] contend that the liver is an important site for feed intake control. Outside the central nervous system, feed intake is regulated by both the gastrointestinal tract and the liver. Intrahepatic infusion of glucose, lysine or lipids decreased feed intake. Interestingly, these effects vary with the strain of bird. Intrahepatic infusion of glucose decreased feed intake in laying hens, but not in broilers [61]. Similarly, infusion of lipids decreased feed intake in Leghorns, but not broilers [62]. 

It has been reported that feeding diets with high energy to protein ratios increased fat synthesis and deposition in broilers [63,64,65,66]. On the other hand, feed withdrawal can cause alterations in fat metabolism, as well as fat content and fatty acid composition of the liver and its size [65]. Effects of feed withdrawal on liver size and fat content have been documented in early studies. Imaizumi et al. [67] found an increase in the concentration of stearic and arachidonic acids and decrease in the concentration of palmitic and linoleic acids in the liver of rats after overnight feed withdrawal. Leveille et al. [68] reported that lipogenesis decreases after 2 h of feed withdrawal in growing chickens. Bartov [64] reported that effects of feed withdrawal on reducing liver size and fat content were significant after 10 h and increasing the duration of feed withdrawal to 24 h had a further effect. They also observed the same pattern of fatty acid composition in birds subjected to feed withdrawal for 10 or 24 h. Bartov [65], showed that feed withdrawal markedly affected the composition of liver fatty acids, with increase in the concentration of stearic, linoleic and arachidonic acids and decreases in those of palmitic and oleic acids. These changes in the pattern of fatty acids might be due to a relative increase in the phospholipid fraction, which contains high concentration s of arachidonic and stearic acids and low concentration of oleic acid, compared to the triglyceride fraction [69,70]. In contrast, Shapira et al. [71] stated that liver fatty acid composition of young broilers was not affected by 24 h of feed withdrawal. Rosebrough et al. [72] feed-restricted male broiler chicks from 6 to 12 days of age and reported heavier relative weights of liver for the restricted birds on 14, 16, and 18 days of age, compared with the control group without restriction. Fontana et al. [73] observed no significant differences in liver weight between feed-restricted birds (7 days of restriction) and ad libitum controls at 28 and 49 days of age in their first experiment. However, in the second experiment of the same study, they reported that feed-restricted birds (for 7 days) showed higher relative liver weight compared to those fed ad libitum. However, there were no significant differences in relative liver weights between 4 days feed restriction and ad libitum feeding. Fontana et al. [73] also reported that bird sex and, dietary concentrations of fat, protein and total sulphur amino acids had no effect on liver weights. Feed restriction is often associated with greater water consumption [74,75] and hydration of the digesta, which may lower digesta viscosity and, increase the absorption of lipids and lipid-soluble vitamins (vitamin D3) causing accumulation of fat in the liver. Zubair and Leeson [76] observed some evidence for adaptation by restricted re-fed broilers, such as relative enlargement of digestive organs, especially the gizzard, crop, pancreas and liver. But this finding is not supported by Susbilla et al. [77], who applied feed restriction of 50 and 75% of ad libitum intake to broiler chicks from 5 to 11 days of age and found no differences in the proportional liver weight.

Overall, the enlargement of the liver observed during re-feeding after feed restriction in many studies may be an adaptation to enable the birds to increase the rate of fat deposition [76]. Higher activities of hepatic lipogenic enzymes during re-feeding, following suppression by early feed restriction, lend support to this premise [72]. 

## 7. Effects of Anti-Nutritional Factors

Many feed ingredients that are commonly used in poultry feed formulations contain anti-nutritional factors. These factors interfere with the utilisation of nutrients in a variety of ways including binding nutrients, complexing and inactivating digestive enzymes and, inducing changes in the morphology and pathology of gut and organs, thereby adversely affecting the digestive efficiency, health and productivity of the animal [78]. The effects of these factors on the animal productivity and nutrient digestibility are well documented, but the influence on the liver, the detoxifying organ in the body, has not been adequately investigated. Ortiz et al. [79] found histological lesions in the ileum and liver of the birds and rats fed dried tannin extract from faba bean (*Vicia faba*). In their study, degeneration of the hepatocytes was observed. Emiola et al. [80] reported that the anti-nutritional factors in raw and processed kidney beans reduced the relative weights of the liver in birds. Histology of the liver showed an extensive coagulative necrosis, congestion of sinusoid and extensive degeneration of the hepatocytes. Liver contains high concentrations of inositol [81], largely synthesised from glucose [82]. Myo-inositol has been reported to be a hepatic lipid exporter. Hayashi et al. [83] observed an increase in triglyceride accumulation in the liver of rats fed highly saturated fat along with an inositol-deficient diet. However, when highly unsaturated fats were fed with an inositol-deficient diet, this effect was not observed. The results of several studies suggest that inositol plays an important role as a transporter of dietary fats [82,84]. The mechanisms by which inositol reduces fat mass is not yet known. However, Croze et al. [85] suggested that reduced fatty acid synthase activity in adipose tissue could be the possible mechanism.

Liu et al. [86] found that the phytase addition in broiler diets can modify serum and liver lipid profiles, as well as the mRNA expression of leptin. These data imply that phytate, an antinutrient that binds phosphorus and lowers its availability, influences lipid metabolism and deposition in the bird. Katayama [87] investigated the effect of dietary sodium phytate and found an increase in liver weight, total lipids, triglyceride and cholesterol content of the liver and decrease activities of hepatic lipogenic enzymes including Nicotinamide Adenine Dinucleotide Phosphate Hydrogen (NADPH)-generating enzymes. Viveros et al. [88] reported that lowering of non-phytate phosphorous content as well as supplemental phytase increased the relative liver weight in broilers. Phytase supplementation in poultry diets has been reported increased the hepatic concentration of coenzyme Q10 (ubiquinone), suggesting an improvement in the antioxidative status of phytase-fed birds [89,90]. Sharma et al. [91] added four different doses of phytase (0, 500, 1000, 1500 FTU/kg) to broiler diets and reported no effect of phytase dose on the relative weight of the liver.

## 8. Effect of Dietary Fibre and Exogenous Carbohydrases

Dietary fibre is divided in two categories, namely water insoluble fibre (IDF) and soluble dietary fibre (SD). Insoluble dietary fibre sources such as oat hulls, sugar beet pulp, cellulose and soybean hulls, which are used as diluents in poultry diets [92], have negative effects on feed intake and nutrient digestibility [93,94], but these effects are dependent on the inclusion level and type of fibre. In recent years, low level inclusion of IDF has been reported to have positive influence on growth performance, gut health, gut microbial profile and gizzard development, and increase the secretion of hydrochloric acid, bile and digestive enzymes and consequently improve nutrient digestion [95,96,97,98,99,100]. Akiba and Matsumoto [100] stated that that IDF has important roles in lipid metabolism through changes in lipid synthesis in the liver and enzyme activities in the adipose tissue. In an earlier study, Akiba and Matsumoto [101] found that the liver weight was depressed by the inclusion of cellulose. Liver lipid content was also reduced by cellulose, rice hull and alfalfa meal. It was suggested that fibre is able to reduce liver lipid deposition and plasma lipid content. However, these results did not reveal whether the reduction in liver lipids was caused by fibre content or rather reduced energy intake. Woyengo et al. [102] reported that reduced weight gain because of increased IDF (from canola meal) in broiler diets can partly be attributed to increased liver metabolic activities. Hetland et al. [95] found an increase in bile secretion when gizzard activity was stimulated by the addition of oat hulls in broiler diets. The increased metabolic activities of the liver can result in increased utilisation of various nutrients including amino acids, minerals and vitamins for maintenance at the expense of tissue deposition [103,104] and lead to reduced growth performance. In contrast to these results, Mossami [105] found that weight and lipid content of the liver were not affected by graded inclusions of oat hulls (0, 20, 40, 60 and 80 g/kg) in broiler diets.

Based on the above, it can be concluded that the effect of IDF on liver metabolism and size depends on feed intake and inclusion rate of IDF. Lower feed intake by feeding IDF can cause the higher metabolic activity of the liver and increase its size. 

Cereals such as wheat, barley, oats, rye, and their by-products contain a high proportion of partly soluble dietary fibre polysaccharide residues which are called non-starch polysaccharides (NSP) [106]. Non-starch polysaccharides can increase the viscosity of the digesta and inhibit effective contact between the digestive enzymes and their corresponding substrates, leading to significant modifications of the structure and function of intestine and digestive organs [107]. To overcome the negative effect of NSP on digestive tract exogenous carbohydrases can be used in the diet [108]. Published data on the effects of exogenous enzymes on the liver size and metabolism in broilers have been contradictory, possibly due to differences in enzyme type and composition of basal diets. For example, Brenes et al. [109] reported that the addition of an enzyme preparation containing mainly ß-glucanase and xylanase to a barley-based diet reduced the relative weight of liver but had no effect when included in wheat-based diets. Wu and Ravindran [110] reported that xylanase supplementation had no effect on the relative weights of the crop, proventriculus, gizzard, pancreas, liver and heart of birds fed wheat-based diets. Lázaro et al. [111] found that combination of xylanase and β-glucanase supplementation to rye-based diets did not influence the relative weight of the liver. Zhu et al. [106] similarly found that supplemental enzymes (xylanase, β-glucanase, and α-amylase) had no effect on the liver size in broilers fed maize-based diets at 7, 14 and 21d post-hatch. 

Józefiak et al. [112] observed an interaction between grain type and enzyme supplementation on liver weight. ß-glucanase supplementation increased liver weight in barley-based diets but decreased it in oat-based diets. Sayyazadeh et al. [113] fed broilers with different cereal sources (maize, barley and wheat, individually or in combination) either without or with enzyme addition. It was reported that weight gain and feed intake were influenced by the dietary treatments, but liver weights were unaffected. In contrast, Al-Marzooqi and Leeson [114] reported that the relative weight of the liver was greater following the addition of pancreatic enzyme at day 21 in broilers fed maize-based diets. However, the enzyme had no effect on liver weight at day 42. They suggested that the increase in liver weight at day 21 was reflective of increased metabolic activity relating to increased fat utilisation. 

Wang et al. [108] fed wheat-based diets supplemented with 0, 200, 400, 600, 800, or 1000 mg/kg of an enzyme preparation (xylanase and β-glucanase)) to broilers and reported linear decreases in the relative liver and pancreas weights with increasing enzyme supplementation at day 21 and 42 of age. To adapt to the structural and functional changes of the intestine and organs in birds fed diets containing viscous grains, the activities of the intestinal secretory mechanisms are reported to increase [107]. This may lead to increases in the size of the GIT, pancreas, and liver. Brenes et al. [109] indicated that the increased size of GIT organs in birds fed viscous grains could be an adaptive response to an increased need for enzymes. 

Emadinia et al. [115] examined the influence of wet feeding and multi-enzyme supplementation to wheat-based diets and reported that wet feeding increased relative liver weights compared to dry feeding, but enzyme supplementation had no effect. They suggested that higher liver weight with wet feeding might be due to the viscosity reduction, consequently increasing nutrient availability and metabolism in the liver. 

Parsaie et al. [116] stated that the liver is a site of detoxification and nutrient metabolism, thus it is suggested that the liver size is dependent on the amount of work it does. Brenes et al. [109] stated that, after intestinal absorption, the liver is the major site of short chain fatty acids metabolism, especially of propionic and butyric acids. Józefiak et al. [117] suggested that the liver size is influenced by the gut microbiota profile and their fermentation products.

The above discussion indicates that, when exogenous enzymes are added to a wheat-based diet, a greater proportion of NSP may be hydrolysed and may attenuate the secretory function of the responding organs and GIT leading to decreased organ sizes. However, high microbial activity due to the high digesta viscosity may not be the only factor affecting liver weight. Iji et al. [118] reported that viscous sugars increased the weight of the small intestine but not of the the proventriculus, gizzard and liver in broiler chickens. 

## 9. Effect of Feed Structure and Whole Grain Feeding

Although the effects of pelleting on bird growth and nutrient digestibility have been studied extensively [119,120,121,122,123], studies regarding its effect on liver metabolism and size are limited. However, it has been reported that fat synthesis and deposition was increased by feeding crumbled or pelleted diets to broilers [124,125]. Bartov [65] reported that in birds fed pelleted diets, abdominal fat pad, liver fat content and liver size increased compared to those fed mash diets. Rezaeipour and Gazani [126] found that that relative liver weight was greater in birds fed pelleted diet compared to birds fed mash diets. Pelleting may not benefit nutrient digestibility in some cereals, but it increases feed intake and subsequently total nutrient intake compared to the mash diets [119]. Increased size and fat content of liver in pelleted fed birds compared to mash-fed birds may partly be explained by higher nutrient availability for hepatocytes. 

Perez-Maldonado et al. [127] observed that birds fed steam-pelleted (70–80 °C) diets gained more weight and consumed more feed than those fed cold-pelleted (45 °C) diets. However, liver weight was not affected by conditioning temperature. Preston et al. [128] found that there were no differences in liver weight between mash, cold-pelleted, steam-conditioned/pelleted, wet mash and whole wheat diets. Similar results were reported by Scott [129], who found that there were no effects of feed form (crumble vs. mash) on the relative liver weight at 12, 20 or 34 days of age. Gonźalez-Alvarado et al. [130] fed birds with maize or rice-based diets with two heat-processing treatments of the cereals (raw and cooked) and observed no differences in relative liver weight among dietary treatments. Mirghelenj and Golian [131] found no effect of feed form (mash, pellet and crumble-pellet) on liver, pancreas and heart weights at day 42. Similar finding reported by Ghobadi and Karimi [132]. Senkoylu and Dale [53] showed that pelleting diet with high-oil sunflower meal did not affect the weight or lipid content in the liver of broilers.

The influence of feed particle size on gizzard development is well documented [97,133]. However, no study has investigated the effect of feed particle size on liver metabolism and function. Benedetti et al. [134] found that birds fed coarse maize dies had 15% higher liver weight compared to those fed fine maize diets. Similar results have been reported in turkeys by Favero et al. [135], who found that the diet containing coarse maize particles promoted an increase in relative liver weight compared with those containing fine and medium maize particles. In contrast to these results, Rezaeipour and Gazani [126] reported that feed particle size had no effect on liver weight in broiler chickens. 

Overall, these results suggest that pelleting may not affect liver size but increase the metabolism and transfer rate of nutrients from the liver to other tissues. It is also tempting to suggest that the metabolic activity of the liver might be enhanced by feeding coarse particles, but further research will be needed to confirm this hypothesis. 

A recent development in poultry nutrition is the increased use of whole grains as a mean of improving gizzard development, nutrient digestion and gut health [136]. In whole grain studies, the focus had been on the effects on bird performance and gizzard development, but effect on liver metabolism and function has often been overlooked. In several studies, however, liver size has been measured (Table 3). Nahas and Lefrancois [137], examining the effect of whole wheat inclusion (100–200 and 200–350 g/kg replacing maize during days 7–21 and days 22–38, respectively) on the performance and organ weight of broiler chickens, reported that weight of organs (crop, pancreas, proventriculus, liver, heart and gizzard) was not affected by the inclusion of whole wheat. Wu and Ravindran [110] reported that whole wheat inclusion post pelleting decreased the relative weight of the liver compared to those fed diets containing ground wheat. In a subsequent study, Ravindran et al. [10] found that the relative weight of liver was heavier at day 14 with whole wheat feeding compared to ground wheat-based diet. They reported that the biological significance of this finding is unclear. Singh et al. [138] fed birds with five diets containing 600 g/kg of ground maize or 150, 300, 450, and 600 g/kg of whole maize replacing (w/w) ground maize. A quadratic response was observed for the relative weight of liver, with the weight decreasing at 150 g/kg inclusion, plateauing with further inclusion and then increasing from 450 to 600 g/kg inclusion. Svihus et al. [139] found that jejunal bile concentration were higher in birds fed whole wheat (post-pelleting) diets. They reported that pre-pelleting inclusion of whole wheat did not affect bile concentration in the jejunum or gizzard. They proposed that the enhanced feed value observed with whole wheat may be associated with modulation of digestive processes resulting in increased pancreatic and liver secretion and activity.

## 10. Effect of Nutrient Density

As the liver is responsible for bile formation which is used in fat digestion [18], it is expected that increasing dietary nutrient density (with high fat levels) increases bile formation due to its role as a fat emulsifier. As mentioned above, the liver is involved in the processes of glycolysis (using glucose as input) and beta oxidation (using fatty acids as input) for energy yielding purposes [142]. In Scott [129] study, two diet densities were applied for the starter and grower diets; high (ME 3170/3200 kcal/kg; protein 25.1/21.0%) or low (ME 3100/3060 kcal/kg; protein 23.5/19.5%) in starter/grower diet, respectively. He reported that increasing diet density resulted in lower liver weights compared to a regular diet density in broilers from 12 days of age onwards. Lamot et al. [143] used dose-response design consisting of five dietary fat concentrations (3.5, 7.0, 10.5, 14.0, and 17.5%) through soybean oil inclusion. Amino acids, minerals, and the premix were increased at the same ratio as dietary fat. They found that both the absolute and relative liver weights decreased as dietary nutrient density increased at 7 days of age. In young broiler chickens, protein synthesis can use almost half of the retained energy [143,144]. High energy requirement for growth and protein retention may result in increased glycolysis in the liver and most of it will likely be used as fuel for growth, thus partly explaining a reduced liver weight in starter chickens fed high density diets. Fatty acids that are not used for energy yielding purposes through β-oxidation, can be deposited as triglycerides in the liver. But in young broiler chickens, due to the relatively high protein retention rate and synthesis, it is expected that fatty acids are (in addition to glucose) mainly used for energy yielding purposes [144]. Therefore, reduced storage of fatty acids in the liver may explain to some extent a reduced liver weight.

It can be concluded that increased bile formation for fat emulsification may not result in increased liver weight. Lower liver weight in broilers fed high density diet is due to its metabolic function to facilitate the increased growth rate.

## 11. Liver Metabolic Disorder

Dietary fatty acids in birds are secreted into the portal blood system as portomicrons. Due to the direct entry into the portal blood system, portomicrons pass through the liver before they reach the rest of the circulation. Such a feature predisposes birds to fat deposition in the liver [15] and also exposes the liver to various infectious agents and toxins. The disorders of liver have far reaching consequences on the performance and health of birds. The resultant pathological changes are expressed hepatosis, necrosis, fatty liver, hepatitis and cholangitis. The pathology and lesions of liver are specific to the agent, and frequently used to identify the exact cause.

Disorders of lipid metabolism are a group of metabolic diseases that occur in all animals, including chickens. Fatty liver haemorrhagic syndrome (FLHS) is the most important metabolic disorder in poultry that involve the occurrence of fatty deposits in the liver. 

### Fatty Liver Haemorrhagic Syndrome (FLHS)

Fatty liver haemorrhagic syndrome is a significant disease of caged layer hens. It is observed during high production periods in laying hens. It is characterised by excessive accumulation of fat in the liver and abdominal cavity, liver rupture and haemorrhage, and sudden death [145]. Necropsy of dead hens with FLHS reveals that the birds have enlarged and pale livers. Affected birds also have pale combs. The liver cells are distended with fat vacuoles and different size haemorrhages. The kidneys are also pale and swollen. Large amounts of yellow and partially liquid fat are present in the abdominal cavity and around the viscera [146]. Analysis of the livers in affected hens shows remarkably high fat concentrations. These generally exceed 40% dry weight and are sometimes above 70% and largely result from increases in the number of triglycerides [147,148]. The actual cause of the disease is still unclear, and the first sign is often an increase in mortality in the flock [149]. More details about the FLHS can be found in the comprehensive reviews by Butler [146], Whitehead [149] and Meijering [150]. Several factors can cause increased deposition of fat in the cells of the liver. 

Fatty liver syndrome occurs when high producing hens are in over-supply of energy or a positive energy balance, but the presence of these conditions does not guarantee the appearance of FLHS [96,146]. Butler [151] suggested that excess fat in the liver arises mainly from increased lipogenesis rather than from dietary lipids. Although there has been no constant association with a particular type of diet, some studies have indicated that high energy diets, especially maize or wheat diets produce higher incidences of FLHS [146,152,153,154]. Jensen et al. [155] also stated that the amount of fat deposited in the liver is influenced by the cereal used as the basis of the diet. For instance, with iso-caloric diets based on maize, wheat or barley, the incidence of subclinical FLHS and liver lipid concentration was observed to decrease in that order [153]. Similarly, the inclusion of such ingredients as fermentation residues, wheat bran or alfalfa have been found to significantly depress liver lipid concentrations [156]. Olomu et al. [157] reported that the incidence of liver FLHS tends to increase with the amount of rapeseed meal in the diet due to the erucic acid or other toxic metabolites which can affect the strength of the connective tissue in the liver [158,159]. Earlier finding by Hemsley [160] and Payne et al. [161] showed that the syndrome was due to biotin deficiency. Frigg [162] reported that very little of the biotin present in wheat, and some other cereals, is available to the chicken. Therefore, diets based on large proportions of these feedstuffs contain sub-optimal concentrations of available biotin and hence require to be supplemented. 

There is evidence that among the mycotoxins, aflatoxin can cause fatty livers in laying hens [163]. Other fungal metabolites are known to possess oestrogenic activity and it is possible that their presence may cause the occurrence of the FLHS. Dietary modification can be used to prevent or treat FLHS. Substituting carbohydrate with supplemental fat might be beneficial. 

Hepatic steatosis prevalence in caged birds is believed to be associated with the lack of exercise combined with a high feed intake in this housing system [164,165,166,167]. Shini et al. [167] demonstrated that hens in cages had significantly higher body weights than barn and free-range systems birds. This presumably causes a positive energy balance induced from a lack of exercise due to restricted space in cages. 

Temperature is an important environmental factor that affects the incidence of FLHS in caged birds. The energy requirement of the hen, which has already been reduced by the restriction of exercise imposed by the cage or pen, is reduced further by a high environmental temperature which discourages movement and reduces heat loss. Under these circumstances certain hens are unable to reduce their food consumption sufficiently to avoid a positive energy balance [168], possibly because the diet does not contain enough fibre to satisfy appetite. Ivy and Nesheim [169] found that occurrence of the syndrome was most frequent during the summer months. Studies showed that stress related to the high temperature and humidity of the environment affect the liver of hens and predisposes them to FLHS [170,171]. Clark and Das [172] found that in heat stressed birds, hepatic tissue has a fatty appearance and pale with small areas of haemorrhage similar to FLHS. However, when birds were kept at 21 to 38 °C, the liver weight did not change. Lee et al. [173] observed that keeping temperature controlled in the thermo-neutral zone does not decrease the incidence of FLHS suggesting that stress is more important factor than ambient temperature.

The association of FLHS with birds that have a high level of egg production may thus be a possible indication that there is a hormonal involvement in the FLHS disorder. The administration of oestrogens to immature fowls has produced hyperlipaemia and liver lipid accumulation by increasing lipogenesis [174]. Oestrogens influence lipid synthesis which is required for the yolk [175]. Studies on laying hens have shown that high oestrogen levels result in increased feed intake and subsequently in a positive energy balance. High producing birds within a flock are most often affected by FLHS [176], most probably due to a relationship between energy metabolism and hormone levels during egg production. Polin and Wolford [177] indicated that the haemorrhage score in liver was markedly increased when excess energy intake was combined with exogenous oestrogen treatment. 

An early study by Couch [178] showed that heavy and higher producing breeder hens are more susceptible to FLHS. Stake et al. [179] also found that compared to White Leghorn hens, Rhode Island Red hens are more sensitive to experimental induction of FLHS. Moreover, a strain of single comb White Leghorn laying hens (UCD-003) has been shown to be highly susceptible to FLHS [180]. The tendency to leanness or fatness in birds is related to fundamental metabolic differences in the partitioning of nutrients, in which hormones are likely to play a major role [181]. 

## 12. Conclusions

The liver is one of the most dynamic organs in birds, performing a diverse array of functions. It is responsible for most of the synthesis, metabolism, excretion, and detoxification processes in the body. The liver plays an important role in digestion and metabolism, regulating the production, storage, and release of carbohydrates, lipids and proteins. Since nutrients are synthesised or stored in liver and then transported in the plasma to the storage sites, the greater mass of the liver is considered as a positive indicator and associated with higher metabolic activity. However, a larger liver with higher metabolic activity may also have higher energy expenditure. Increased liver size in birds fed pelleted diets may partly be explained by higher availability of nutrients in hepatocytes due to higher feed intake. Optimisation of the function and overall health of the avian liver might be favourably modified by feeding techniques such as use of exogenous enzymes, dietary fibre inclusion, coarse particles and whole grain feeding, but these aspects require further study.

## Figures and Tables

**Table 1:** Liver weight and proximate analysis of liver in broiler chickens fed maize-based diets (d 21) ^1^.

Fat Source	Weight (g/kg BW)	DM (g/kg)	Protein (g/kg)	Ash (g/kg)	Fat (g/kg)
Soybean oil	26.9	241	690	59	125
Tallow	26.3	234	688	60	130

^1^ Each value represents the mean of 12 birds.

**Table 2:** Concentration of bile acids in the bile of the chicken and duck (mg/g).

Bile Acid	Chicken	Duck
Cholic acid	9.6 ± 0.5	45.2 ± 2.3
Chenodeoxycholic acid	25.2 ± 2.2	28.2 ± 1.6
Ursodeoxycholic acid	n.d.	43.5 ± 2.1
Deoxycholic acid	n.d.	31.6 ± 1.9
Lithocholic acid	68.7 ± 2.1	37.5 ± 2.1
Taurocholic acid	152.6 ± 3.1	16.8 ± 1.5
Taurochenodeoxycholic acid	n.d.	97.5 ± 3.4
Taurolithocholic acid	35.9 ± 0.6	n.d.
Glycolithocholic acid	228.4 ± 1.6	n.d.

n.d. = not detected; Source: Yeh and Hwang [34].

**Table 3:** Influence of whole grain inclusion on weight gain and relative liver weight (g/kg body weight) in broilers (% of changes relative to control).

Reference	Age (day)	Grain Type	Whole Grain Inclusion (g/kg)	Weight Gain	Liver Weight
				% Changes	
[137]	7–38	Barley	100 ^1^, 150 ^2^	+0.6	+6.5
			150 ^1^, 150 ^2^	+2.4	+8.4
[137]	7–38	Wheat	100 ^1^, 200 ^2^	+4.2	+7.8
			100 ^1^, 350 ^2^	+5.1	+3.7
			200 ^1^, 200 ^2^	+1.3	+3.7
			200 ^1^, 350 ^2^	+2.0	+9.3
[10]	1–14	Wheat	100	−9.7	+11.9
[140]	21–42	Wheat	300	−3.8	+8.5
[138]	11–35	Maize	150	+3.3	+1.3
			300	+7.0	+16.2
			450	+12.0	+8.3
			600	+8.7	+5.7
[141]	1–10	Sorghum	250	+8.7	+5.7
			500	+4.4	−3.4
			750	+5.6	+8.4
[141]	1–35	Sorghum	250	−0.87	+5.3
			500	−6.5	+5.7
			750	−5.2	+6.0

^1^ Grower phase (day 7–21); ^2^ Finisher phase (day 21–38).
